# Endothelial protein C receptor and thrombomodulin facilitate protease-activated receptor 1 cleavage at Arginine-46 by thrombin and activated protein C

**DOI:** 10.1016/j.jbc.2025.110339

**Published:** 2025-06-04

**Authors:** Indranil Biswas, Mariko Kudo, Alireza R. Rezaie

**Affiliations:** Cardiovascular Biology Research Program, Oklahoma Medical Research Foundation, Oklahoma City, Oklahoma, USA

**Keywords:** thrombomodulin, thrombin, EPCR, APC, signaling, endothelial cells

## Abstract

Protease-activated receptor 1 (PAR1) has two cleavage sites for activation by coagulation proteases (Arg41 and Arg46). The cleavage of Arg46 by either activated protein C (APC) or thrombin leads to cytoprotective signaling; however, neither protease can cleave this site in the absence of their receptors, endothelial protein C receptor (EPCR) and thrombomodulin (TM), respectively. Arg41 is the preferred cleavage site for both proteases in the absence of receptors. The mechanism by which these receptors function as cofactors to catalyze the cleavage of PAR1-R46 by coagulation proteases is not known. Here, we hypothesized that both receptors alleviate inhibitory interactions of the P2-Leu45 residue on the extracellular domain of PAR1 with protease catalytic pockets. To test this hypothesis, we prepared a PAR1-R41A mutant in which P2-Leu45 of the receptor was substituted with a Pro. Both PAR1-R41A and PAR1-R41A-L45P constructs were transfected to PAR1-knockout EA.hy926 endothelial cells lacking or expressing EPCR or TM followed by monitoring the protease activation of receptors by signaling assays. Furthermore, WT or EPCR and TM expressing human embryonic kidney 293 cells were transfected with PAR1 cleavage reporter constructs carrying N-terminal NanoLuc luciferase and C-terminal enhanced YFP tags. Signaling and receptor cleavage assays indicated that both APC and thrombin cleave Arg46 in cells expressing PAR1-R41A-L45P, but not PAR1-41A, independent of their receptors. The catalytic activity of thrombin was >10-fold faster than APC in both assays. These results suggest that EPCR and TM function as cofactors to alleviate inhibitory interactions of P2-Leu45 of PAR1 with target proteases.

Coagulation proteases contribute to the regulation of inflammatory pathways by proteolytic cleavage and activation of protease-activated receptors (PARs) on vascular endothelial cells. Among four identified PARs (PAR1–PAR4), the structure and physiological function of PAR1 have been studied the most. The extracellular domain of PAR1 has two distinct cleavage sites (Arg41 and Arg46), which are specifically recognized and cleaved by coagulation proteases (1), thereby initiating pleiotropic signaling effects under different conditions depending on whether the proteases are free or in complex with their specific cell surface receptors (2). The cleavage efficiency of PAR1 by factor VIIa alone is very slow and may have no physiological significance, but in complex with endothelial protein C receptor (EPCR), factor VIIa can cleave PAR1-Arg41 site on endothelial cells to elicit cytoprotective signaling effects (3). Although activated protein C (APC) is also a poor activator of PAR1, nevertheless, at nonphysiologically high concentrations, it cleaves PAR1-Arg41 to induce proinflammatory effects in cultured endothelial cells (2). However, when APC binds EPCR *via* its N-terminal γ-carboxyglutamic acid (Gla) domain, the protease cleaves PAR1-Arg46 to initiate physiologically relevant anti-inflammatory and cytoprotective signaling responses in vascular endothelial cells, which have been extensively studied in both cellular and animal models (1, 2). Thrombin is the most efficient activator of PAR1, which rapidly cleaves the receptor at Arg41 site to induce proinflammatory signaling in endothelial cells. The high catalytic efficiency of thrombin toward PAR1-Arg41 is due to the existence of a hirudin-like sequence on the extracellular domain of PAR1 (4), downstream of the Arg41 cleavage site, which binds to basic exosite-1 of thrombin, which facilitates a binary protease–receptor complex formation that leads to promotion of the efficiency of the catalytic reaction (5, 6). Interestingly, while thrombin exhibits no catalytic activity toward Arg46 site of PAR1, we recently discovered that when in complex with thrombomodulin (TM) on the surface of endothelial cells, the protease–receptor complex effectively cleaves Arg46 to induce cytoprotective signaling by the same mechanism that is observed for the APC–EPCR complex (2). The cleavage efficiency of PAR1-Arg46 site by thrombin was found to be greater than 10-fold higher than that of APC and that the improvement in the cleavage efficiency paralleled the same extent of improvement in the cytoprotective signaling function of thrombin as analyzed by signaling assays (2). These findings indicated that EPCR and TM receptors function as cofactors to switch both the cleavage and signaling specificity of APC and thrombin, respectively, by unknown mechanisms.

Coagulation proteases exhibit exquisite recognition specificity toward P3–P3′ residues surrounding scissile bonds of their macromolecular substrates and inhibitors. Most often the residues surrounding the P1-Arg scissile bonds play negative regulatory roles in catalytic reactions unless their target proteases are bound to specific circulating protein cofactors and/or cell surface receptors (7, 8). For efficient substrate recognition and catalytic reactions, thrombin prefers a Pro at the P2 position of substrates and inhibitors. While the P2 position of the P1-Arg41 scissile bond of PAR1 is a Pro, however, this residue is a Leu (Leu45) before the P1-Arg46 scissile bond of the receptor. We hypothesize that P2-Leu45 is a nonfavored residue and plays an inhibitory role, impeding the docking of PAR1-Arg46 to the catalytic pockets of either APC or thrombin, accounting for the inability of these proteases to signal *via* the Arg46 cleavage site. However, the interaction of thrombin and APC with TM and EPCR, respectively, overcomes the inhibitory interaction of Leu45 with these proteases. To test this hypothesis, we prepared a PAR1 mutant in which Arg41 was replaced with an Ala and the P2-Leu45 was replaced with a Pro (PAR1-R41A-Leu45P). The characterization of this PAR1 mutant receptor by signaling and cleavage-reporter assays support the hypothesis that EPCR and TM play essential cofactor roles in overcoming the inhibitory interaction of P2 residue of the PAR1 P1-Arg46 scissile bond with catalytic pockets of coagulation proteases on cell surfaces.

## Results

### Cytoprotective signaling assays

**Figure 1:**
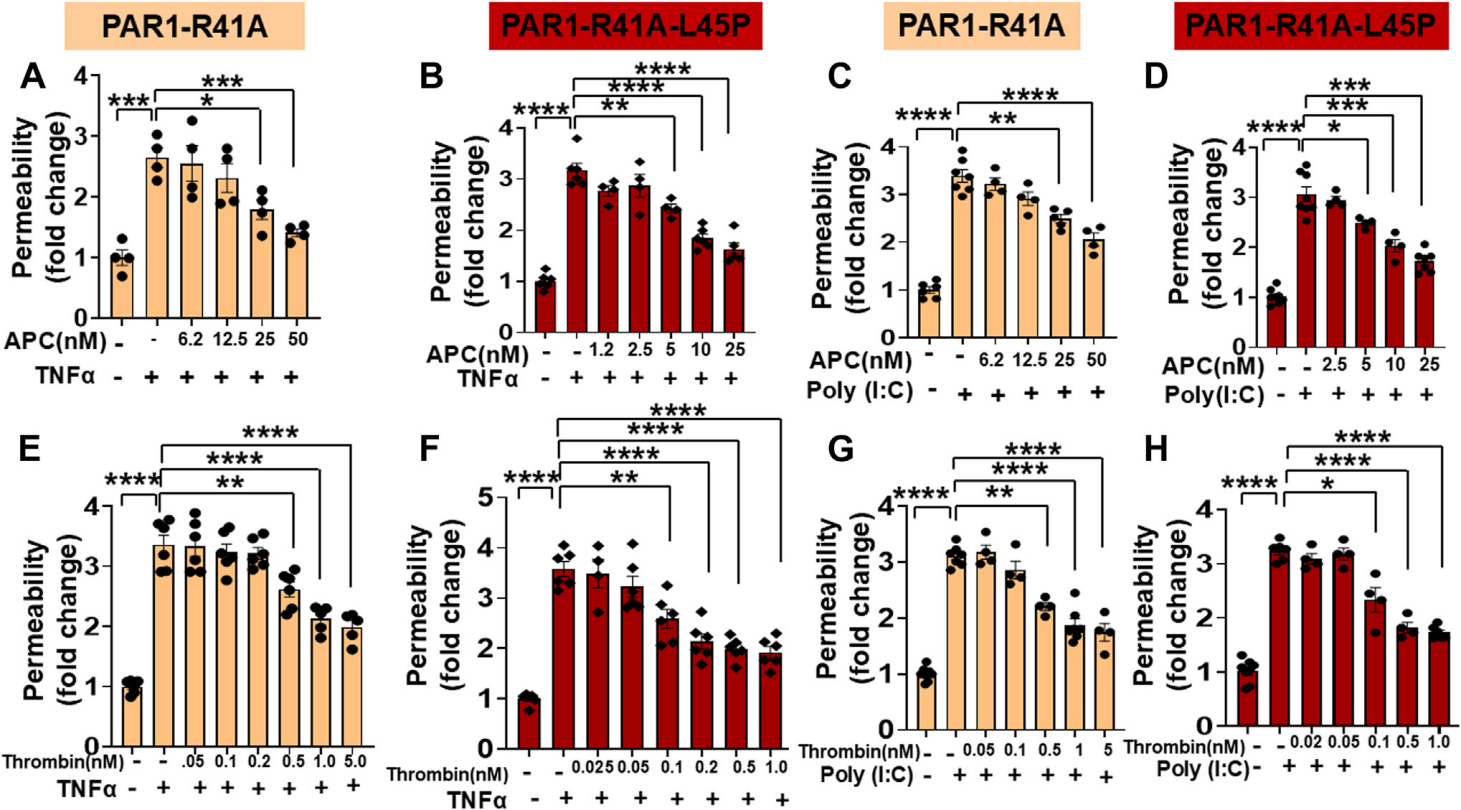
**Analysis of concentration dependence of APC and thrombin in TNFα-mediated permeability assays in PAR1^−/−^ cells expressing PAR1-R41A and PAR1-R41A-L45P**. Confluent PAR1-R41A (*A*) and PAR1-R41A-L45P (*B*) cells were pretreated with increasing concentration of APC for 4 h followed by treatment with TNFα (10 ng/ml) for 16 h. The cell permeability was measured as described in the *Experimental procedures* section (n ≥ 4). *C* and *D*, the same as (*A*, *B*) except that the concentration dependence of the barrier-protective effect of APC (4 h) in confluent PAR1-R41A cells in response to poly(I:C) (10 μg/ml, 16 h) was measured in PAR1-R41A (*C*) and PAR1-R41A-L45P (*D*) cells (n ≥ 4). *E* and *F*, the same as (*A*, *B*) except that the concentration dependence of the barrier-protective effect of thrombin in confluent PAR1-R41A cells (*E*) and PAR1-R41A-L45P cells (*F*) was monitored (n ≥ 6). *G* and *H*, the same as (*E*, *F*) except that the concentration dependence of the barrier-protective effect of thrombin (4 h) in response to poly(I:C) (10 μg/ml, 16 h) was measured in PAR1-R41A (*G*) and PAR1-R41A-L45P (*H*) cells (n ≥ 4). Data are presented as mean ± SEM. *p* Values were determined by one-way ANOVA, followed by a Bonferroni multiple comparison test. ∗*p* < 0.05, ∗∗*p* < 0.01, ∗∗∗*p* < 0.001, and ∗∗∗∗*p* < 0.0001. APC, activated protein C; PAR1, protease-activated receptor 1; TNFα, tumor necrosis factor alpha.

It is well established that APC elicits a barrier-protective effect in endothelial cells in response to proinflammatory cytokines through cleavage of the Arg46 scissile bond of PAR1; however, thrombin cannot cleave this site but rather cleaves the canonical Arg41 site of the receptor to initiate a barrier-disruptive effect independent of cytokines. We recently demonstrated that the Arg46-dependent cytoprotective effect of APC is a receptor (EPCR)-dependent process since inhibiting the interaction of APC with EPCR eliminates the catalytic activity of APC toward Arg46 cleavage and the protease instead cleaves Arg41 site to initiate a barrier-disruptive effect like thrombin (2). Interestingly, we discovered a similar cofactor effect for TM in changing the cleavage and signaling specificity of thrombin and noted that when thrombin binds to TM, the protease–receptor complex cleaves Arg46 site to initiate cytoprotective signaling effects in endothelial cells activated with tumor necrosis factor alpha (TNFα) or other proinflammatory mediators (2). To investigate the mechanism by which the receptors function as cofactors to switch the signaling specificity of the coagulation proteases through cleavage of Arg46, a Leu45 to Pro mutant of PAR1 (PAR1-R41A-L45P) in the background of PAR1-R41A derivative was made, and both constructs were expressed in PAR1^−/−^ EA.hy926 endothelial cells using lentivirus-based expression vectors as described (2). This experimental approach was taken to test the hypothesis that unlike the preferred P2-Pro residue at the P1-Arg41 scissile bond of PAR1, the P2-Leu45 is a poor recognition site for interaction with the active-site pocket of either thrombin or APC for an effective cleavage of the P1-Arg46 scissile bond. The concentration dependence of the APC activity in a permeability assay induced by TNFα indicated that APC, as expected, exhibits a barrier-protective effect in PAR1^−/−^ cells transfected with PAR1-R41A at an optimal concentration of 25 to 50 nM (Fig. 1*A*). The same assay showed that the cytoprotective signaling function of APC was improved about twofold in PAR1^−/−^ cells expressing R41A-L45P mutant of the receptor (Fig. 1*B*). The barrier-protective effect of APC in cells expressing either construct through cleavage of the Arg46 site was recapitulated if instead of TNFα, another proinflammatory mediator, poly(I:C), was used in the permeability assays (Fig. 1, *C* and *D*). We have demonstrated a potent barrier-disruptive effect for poly(I:C) in previous studies (2, 9).

The same assay was used to analyze the concentration dependence of the signaling effect of thrombin in endothelial cells in response to TNFα. Results indicated that thrombin exhibits a more potent barrier-protective effect in PAR1^−/−^ cells transfected with PAR1-R41A with an optimal concentration (5 nM) that was ∼10-fold lower than that observed with APC (Fig. 1*E*). The results further showed that the cytoprotective signaling function of thrombin improved ∼5-fold in PAR1^−/−^ cells expressing the R41A-L45P mutant of the receptor as evidenced by the optimal protective effect occurring at concentrations of 0.2 to 1 nM (Fig. 1*F*). Consistent with results observed for APC, thrombin also induced a barrier-protective effect in cells expressing both PAR1 constructs through Arg46 site in response to poly(I:C) (Fig. 1, *G* and *H*). These results suggest that the cytoprotective signaling function of both APC and thrombin is improved through the cleavage of the Arg46 scissile bond in cells expressing the L45P mutant of PAR1.

### Cytoprotective signaling of APC in PAR1-R41A-L45P cells is independent of EPCR

**Figure 2:**
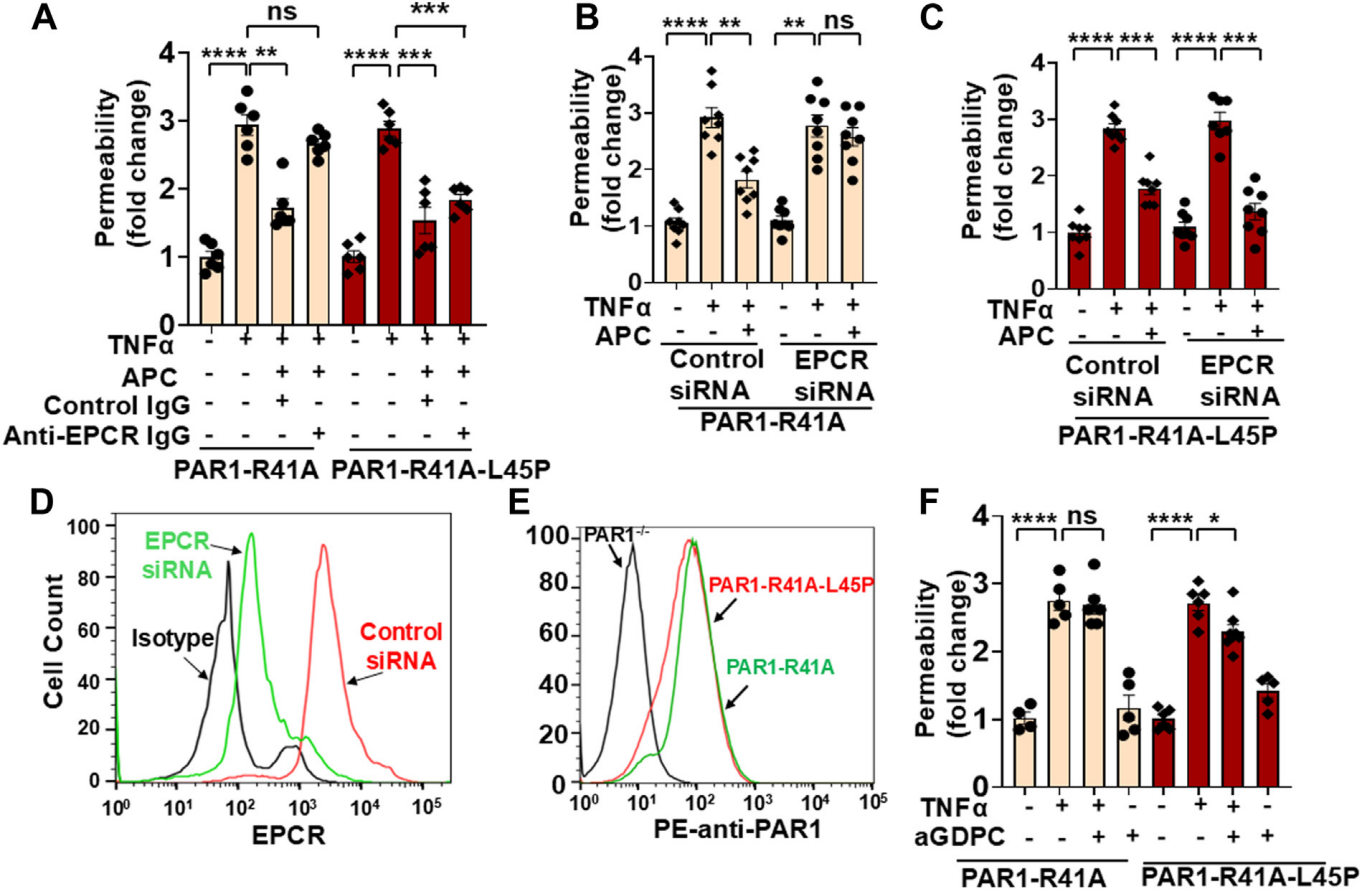
**Analysis of the barrier-protective effect of APC in PAR1-R41A and PAR1-R41A-L45P cells with or without EPCR**. *A*, confluent PAR1-R41A (*orange*) or PAR1-R41A-L45P (*red*) cells were first pretreated with a validated function-blocking anti-EPCR antibody (10 μg/ml) or control IgG (10 μg/ml) for 1 h followed by incubation with APC (25 nM) for 4 h. TNFα (10 ng/ml, 16 h)-mediated endothelial cell permeability was measured as described in the *Experimental procedures* section (n ≥ 5). *B*, PAR1-R41A cells were transfected with either the control siRNA (100 nM) or EPCR siRNA (100 nM). After 48 h, cells were treated with APC (25 nM) for 4 h followed by TNFα (10 ng/ml) for 16 h. The cell permeability was measured as described in the *Experimental procedures* section (n ≥ 6). *C*, the same as (*B*) except that PAR1-R41A-L45P cells were used in the permeability assay. *D*, PAR1-R41A cells transfected with either the control siRNA (*red*) or EPCR siRNA (*green*) for 48 h. Cell surface expression of EPCR was analyzed by flow cytometry using validated anti-EPCR (JRK1535) mouse monoclonal antibody, followed by FITC-conjugated secondary antibody. Normal mouse IgG1 was used as isotype control (n = 3). *E*, cell surface expressions of PAR1-R41A (*green*) and PAR1-R41A-L45P (*red*) were analyzed using PE-conjugated anti-PAR1 (ATAP2) antibody (n = 3). *F*, the same as (*A*) except that the PAR1-R41A (*orange*) and PAR1-R41A-L45P (*red*) cells were pretreated with aGDPC (50 nM) for 4 h followed by treatment with TNFα (10 ng/ml) for 16 h. The cell permeability was measured as described in the *Experimental procedures* section (n ≥ 5). Data are presented as mean ± SEM. *p* Values were determined by one-way ANOVA, followed by a Bonferroni multiple comparison test. ∗*p* < 0.05, ∗∗*p* < 0.01, ∗∗∗*p* < 0.001, and ∗∗∗∗*p* < 0.0001. aGDPC, activated Gla-domainless APC; APC, activated protein C; EPCR, endothelial protein C receptor; PAR1, protease-activated receptor 1; PE, phycoerythrin; TNFα, tumor necrosis factor alpha.

TNFα-mediated permeability assays were used to determine the EPCR requirement for the cytoprotective function of APC in PAR1^−/−^ cells expressing PAR1-R41A and PAR1-R41A-L45P mutants. APC binds EPCR *via* its N-terminal Gla domain, thus a function-blocking anti-EPCR antibody that blocks the interaction of the Gla domain of APC with EPCR was used in the permeability assay as described (2). The results presented in Figure 2*A* indicated that the barrier-protective effect of APC is dependent on its interaction with EPCR; thus in the presence of the antibody, APC exhibited no barrier-protective effect in cells expressing the PAR1-R41A construct. Interestingly, however, the function-blocking anti-EPCR antibody exhibited an insignificant effect on the barrier-protective signaling function of APC in cells expressing the PAR1-R41A-L45P construct (Fig. 2*A*). These results were reproduced if instead of the anti-EPCR antibody, an EPCR-specific siRNA was used to knock down the expression of EPCR in cells expressing PAR1 constructs. While the EPCR-specific siRNA blocked the barrier-protective function of APC in cells expressing the PAR1-R41A construct (Fig. 2*B*); however, it had no effect in blocking the protective function of APC in cells expressing PAR1-41A-L45P (Fig. 2*C*). The effectiveness of the siRNA knockdown of the cell surface expression of EPCR by fluorescence-activated cell sorting (FACS) analysis is shown in Figure 2*D*. The cell surface expression of the mutant PAR1 was also analyzed by FACS analysis using a phycoerythrin (PE)-conjugated anti-PAR1 antibody (ATAP2) as described (2). PAR1^−/−^ cells were used as negative controls. Results indicated that the cell surface expression of both PAR1-R41A and PAR1-R1A-L45P constructs is similar in PAR1^−/−^ cells (Fig. 2*E*). Further support for the EPCR-independent signaling function of APC in PAR1-41A-L45P cells is provided by the observation that activated Gla-domainless APC (aGDPC), which lacks the EPCR-binding Gla domain of APC, has no effect on PAR1-R41A cells in response to TNFα but exhibits barrier-protective effects in PAR1-41A-L45P cells (Fig. 2*F*).

**Figure 3:**
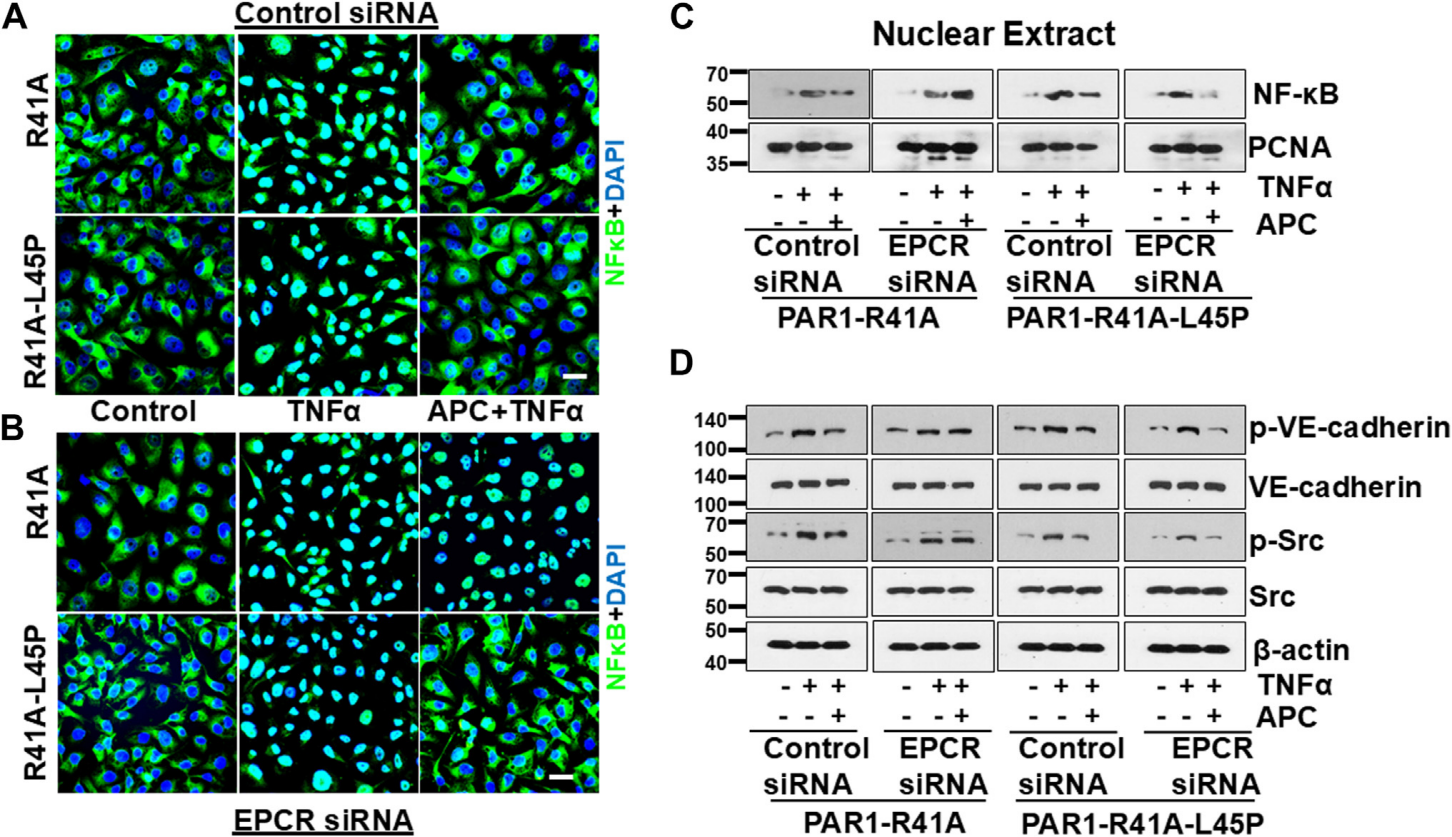
**Analysis of TNFα-mediated NF-κB nuclear localization in PAR1-R41A and PAR1-R41A-L45P cells with or without siRNA-mediated EPCR knockdown**. Cells were transfected with either the control siRNA (100 nM) (*A*) or EPCR siRNA (100 nM) (*B*). After 48 h, cells were treated with APC (25 nM) for 4 h followed by TNFα (10 ng/ml) for 1 h. Cells were then fixed, permeabilized, and stained with anti–NF-κB p65 antibody (rabbit) overnight followed by Alexa Fluor 488–conjugated anti-rabbit antibody. DAPI was used to stain the nucleus. Scale bar represents 20 μm. The images are representative of three independent experiments. *C*, PAR1-R41A and PAR1-R41A-L45P cells were treated with the control or EPCR siRNA (100 nM, 48 h), followed by addition of APC (25 nM, 4 h) and TNFα (10 ng/ml) for 1 h. The nuclear extracts were isolated according to NE-PER Nuclear and Cytoplasmic Extraction kits, and the amount of NF-κB p65 in nuclear extracts was determined by Western blotting. PCNA was used as a loading control for nuclear extracts. *D*, the same as (*C*), except that the cell lysate was prepared after treatment with either control or EPCR siRNA (100 nM, 48 h), followed by addition of APC (25 nM, 4 h) and TNFα (10 ng/ml) for 1 h. Phospho-VE-cadherin (Y658), total VE-cadherin, phospho-Src (Y416), total Src, and β-actin were used for analysis by Western blotting. DAPI, 4',6-diamidino-2-phenylindole; EPCR, endothelial protein C receptor; PAR1, protease-activated receptor 1; PCNA, proliferating cell nuclear antigen; TNFα, tumor necrosis factor alpha.

In agreement with previous results, immunofluorescence analysis revealed that APC inhibits TNFα-mediated NF- NF-κB B activation and its phosphorylation-dependent nuclear localization in endothelial cells expressing both PAR1-R41A and PAR1-R41A-L45P (Fig. 3*A*). Consistent with results of the permeability assays, siRNA knockdown of the EPCR expression abrogated the anti-inflammatory function of APC in PAR1-R41A but not in PAR1-R41A-L45P cells (Fig. 3*B*). This is derived from the data showing that APC has lost its ability to inhibit activation/nuclear localization of NF-κB in EPCR-deficient PAR1-R41A but not in PAR1-R41A-L45P cells (Fig. 3*B*). Western blot analysis of nuclear extracts further supported these results. In the presence of the EPCR siRNA, APC failed to reduce TNFα-mediated NF-κB translocation in PAR1-R41A cells, but this was not true for cells expressing PAR1-R41A-L45P (Fig. 3*C*). Furthermore, Western blot analysis revealed that the EPCR siRNA abrogates the cytoprotective effect of APC in inhibiting the Src-dependent phosphorylation of VE-cadherin in PAR1-R41A but not in PAR1-41A-L45P cells (Fig. 3*D*).

### Cytoprotective signaling of thrombin in PAR1-R41A-L45P cells is independent of TM

**Figure 4:**
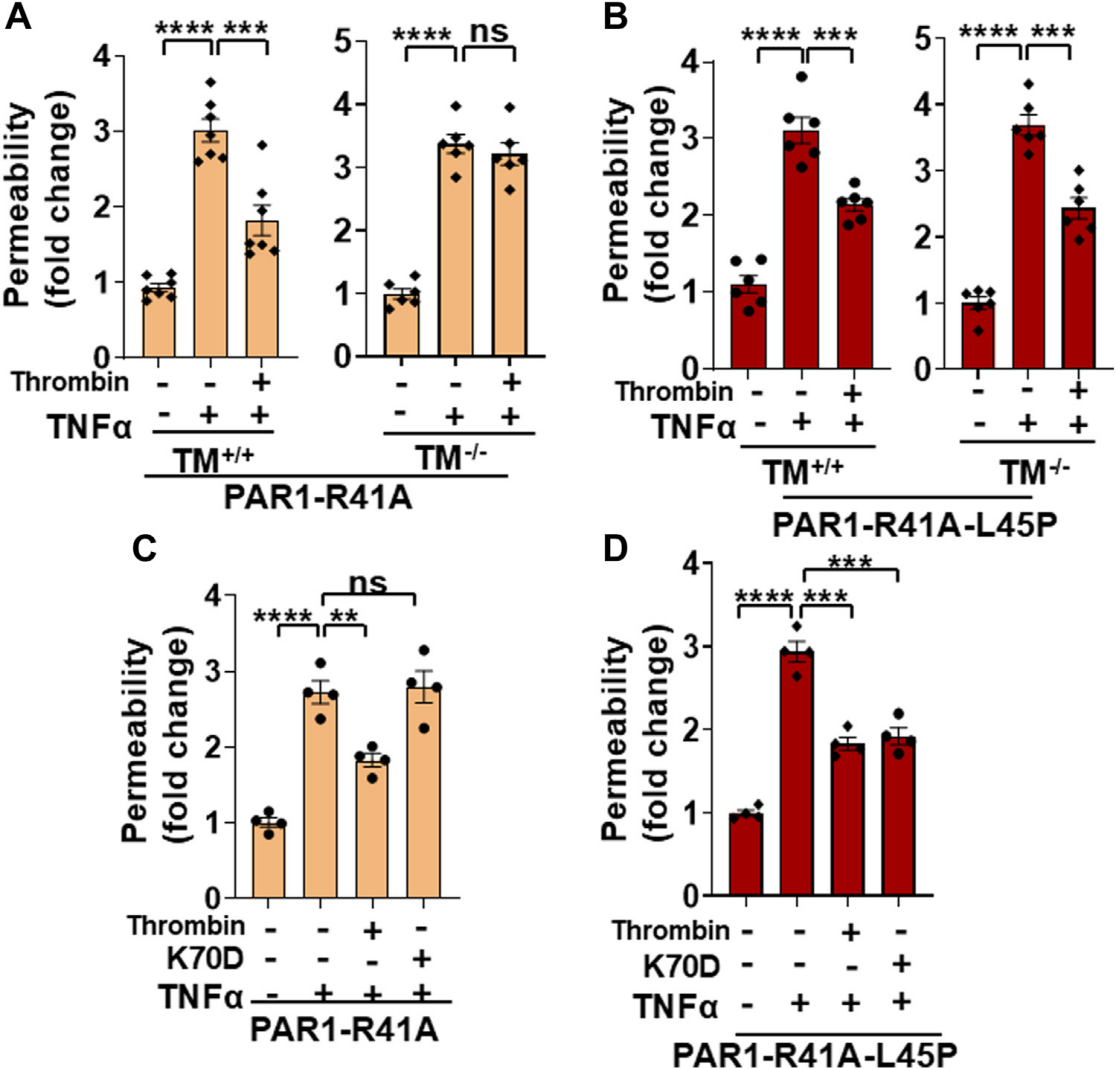
**Analysis of the effect of a WT and an exosite 1 mutant of thrombin in TNFα-mediated permeability assays in TM^+/+^ and TM^−/−^ cells expressing either PAR1-R41A and PAR1-R41A-L45P**. *A*, TM^+/+^ and TM^−/−^ cells expressing PAR1-R41A were pretreated with thrombin (1 nM) for 4 h followed by treatment with TNFα (10 ng/ml) for 16 h. The cell permeability was measured as described in the *Experimental procedures* section (n ≥ 6). *B*, the same as (*A*) except that the barrier permeability effects were measured for PAR1-R41A-L45P cells. *C*, the same as (*A*) except that in the permeability effect for thrombin and thrombin-K70D in TM^+/+^ cells, PAR1-R41A was monitored. *D*, the same as (*C*) except that the permeability was measured for PAR1-R41A-L45P cells (n ≥ 4). Data are presented as mean ± SEM. *p* Values were determined by one-way ANOVA, followed by a Bonferroni multiple comparison test. ∗∗*p* < 0.01, ∗∗∗*p* < 0.001, and ∗∗∗∗*p* < 0.0001. PAR1, protease-activated receptor 1; TM, thrombomodulin; TNFα, tumor necrosis factor alpha.

The same experimental approaches described previously were used to determine whether, like the requirement for EPCR in the cytoprotective signaling through PAR1-R41A, TM is required for the PAR1-dependent barrier-protective function of thrombin in PAR1-R41A-L45P cells. In this case however, instead of blocking the receptor by an anti-TM antibody or knocking down its expression by the siRNA approach, we utilized TM-knockout (TM^−/−^) endothelial cells in which the TM gene has been deleted by the CRISPR–Cas9 methods as described (10). Like the requirement for the cofactor function of EPCR that endows a barrier-protective function for APC through the cleavage of the Arg46 site, TM was required for the barrier-protective function of thrombin in PAR1-R41A cells as evidenced by the lack of a protective effect for thrombin in TM^−/−^ cells expressing this construct (Fig. 4*A*). However, the barrier-protective function of thrombin in response to TNFα in cells expressing PAR1-R41A-L45P was independent of the cofactor function of TM (Fig. 4*B*). Further support for this hypothesis was provided by the observation that a thrombin mutant (K70D), which cannot bind TM because of the substitution of Lys70 of the exosite-1 of thrombin with an Asp (11), did not exhibit a barrier-protective effect in TM^+/+^ cells expressing PAR1-R41A (Fig. 4*C*), but the protective effect with this mutant remained intact in the same cells expressing PAR1-R41A-L45P (Fig. 4*D*). These results suggest that the interaction of thrombin with TM is not required for the cleavage of the Arg46 scissile bond in PAR1-R41A-L45P cells.

**Figure 5:**
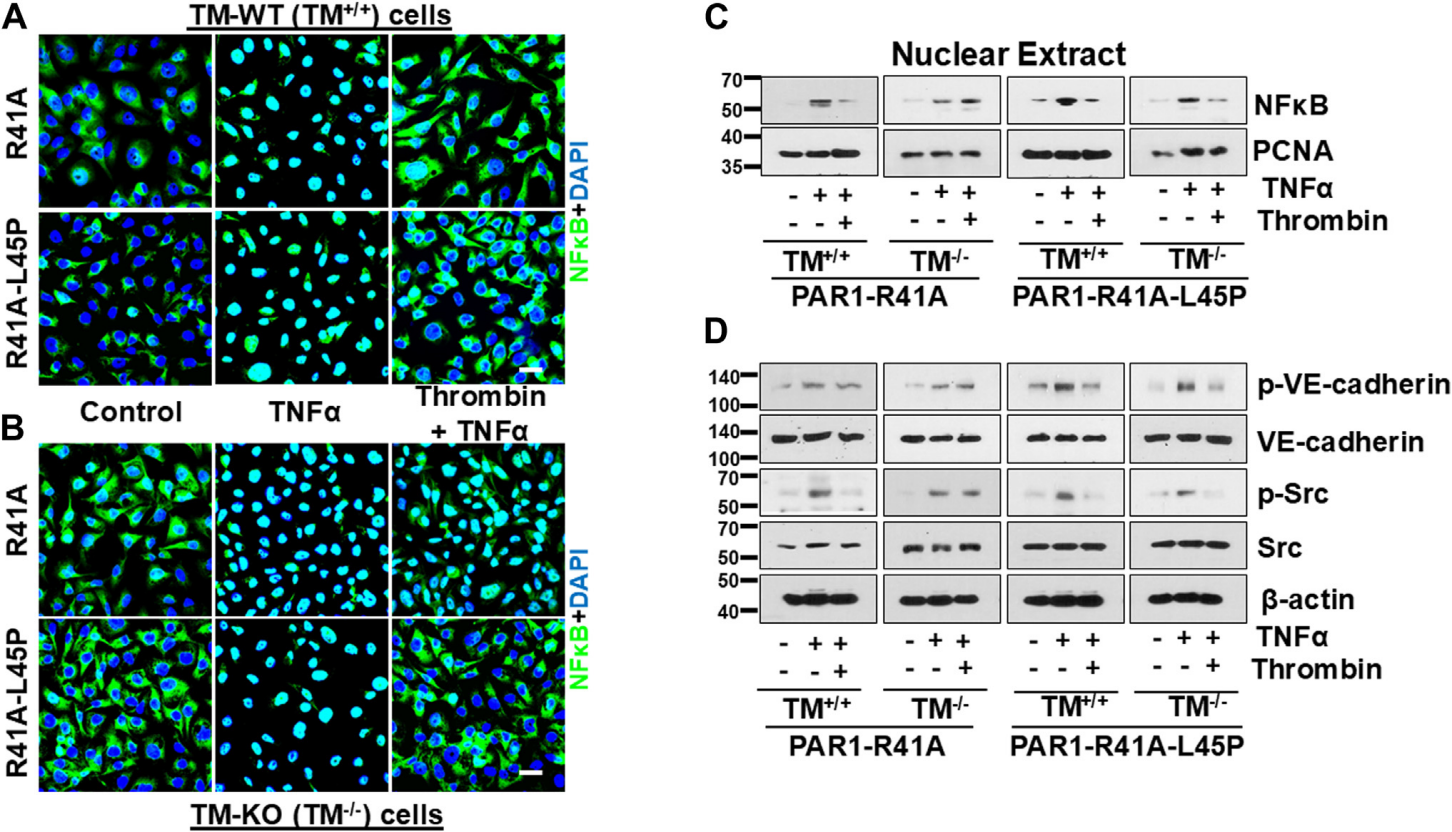
**Analysis of TNFα-mediated NF-κB nuclear localization in TM^+/+^ and TM^−/−^ cells expressing either PAR1-R41A and PAR1-R41A-L45P**. *A*, TM^+/+^ cells expressing either PAR1-R41A or PAR1-R41A-L45P were treated with thrombin (1 nM) for 4 h followed by TNFα (10 ng/ml) for 1 h. Cells were then fixed, permeabilized, and stained with anti–NF-κB p65 antibody (rabbit) overnight followed by Alexa Fluor 488–conjugated anti-rabbit antibody. DAPI was used to stain the nucleus. Scale bar represents 20 μm. The images are representative of three independent experiments. *B*, the same as (*A*) except that TM^−/−^ cells expressing either PAR1-R41A or PAR1-R41A-L45P were used in the experiments. *C*, TM^+/+^ and TM^−/−^ cells expressing PAR1-R41A and PAR1-R41A-L45P were treated with thrombin (1 nM) for 4 h followed by TNFα (10 ng/ml) for 1 h. The amount of NF-κB p65 in nuclear extracts was determined by Western blotting. PCNA was used as a loading control for nuclear extracts. *D*, the same as (*C*) except that the cell lysate was prepared after treatment with thrombin (1 nM) and TNFα (10 ng/ml). Phospho-VE-cadherin (Y658), total VE-cadherin, phospho-Src (Y416), total Src, and β-actin were used for the analysis by Western blotting. DAPI, 4',6-diamidino-2-phenylindole; PAR1, protease-activated receptor 1; PCNA, proliferating cell nuclear antigen; TM, thrombomodulin; TNFα, tumor necrosis factor alpha.

Like results presented in Figure 3 for APC, immunofluorescence analysis indicated that thrombin inhibits TNFα-mediated nuclear localization of NF-κB in cells expressing both PAR1-R41A and PAR1-R41A-L45P (Fig. 5*A*). The anti-inflammatory function of thrombin inhibiting the nuclear localization of NF-κB in PAR1-R41A cells was a TM-dependent process since this effect was abolished in TM^−/−^ cells expressing this construct (Fig. 5*B*). However, thrombin inhibited TNFα-mediated NF-κB activation in TM^−/−^ cells expressing PAR1-R41A-L45P, suggesting that the anti-inflammatory effect of thrombin through cleavage of Arg46 is independent of TM if the P2-Leu45 was substituted with a Pro in the PAR1-R41A construct. Western blot analysis of the nuclear extracts further supported these results as thrombin did not reduce TNFα-mediated NF-κB levels in nuclear extract of TM^−/−^ cells expressing PAR1-R41A, but thrombin inhibited TNFα-mediated NF-κB levels in both TM^−/−^ and TM^+/+^ cells expressing PAR1-R41A-L45P (Fig. 5*C*). Similarly, the cytoprotective effect of thrombin inhibiting the Src-dependent phosphorylation of VE-cadherin in PAR1-R41A cells required TM, but it was independent of the receptor in PAR1-41A-L45P cells (Fig. 5*D*). Taken together, the results presented in Figure 2, Figure 3, Figure 4, Figure 5 clearly indicate that interactions of APC and thrombin with their cell surface receptors, EPCR and TM, respectively, overcome the inhibitory effects of Leu45 of PAR1 in cytoprotective signaling assays, indicating that these receptors function as cofactors to facilitate the docking of the Arg46 scissile bond of PAR1 into the catalytic pockets of these proteases.

### Requirement for **β**-arrestin2 in cytoprotective signaling by APC and thrombin

**Figure 6:**
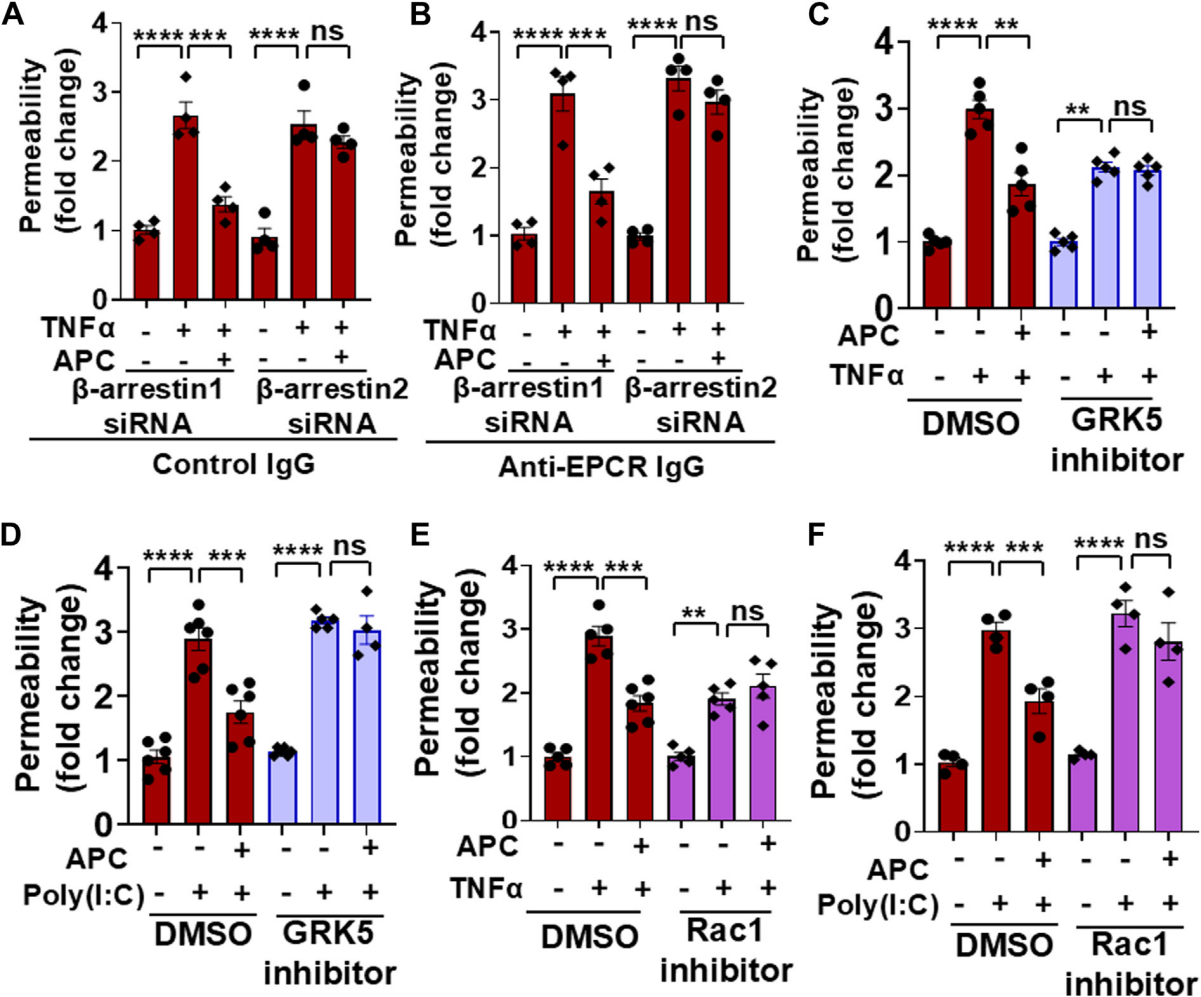
**Analysis of β-arrestins requirement for the cytoprotective APC signaling in PAR1-R41A-L45P cells**. PAR1-R41A-L45P cells were transfected with either β-arrestin1 or β-arrestin2 siRNA. After 48 h, cells were incubated with control IgG (10 μg/ml) (*A*) or the function-blocking anti-EPCR antibody (10 μg/ml) (*B*) for 1 h followed by incubation with APC (25 nM) for 4 h. Then cells were treated with TNFα (10 ng/ml) for 16 h, and the permeability was measured as described in the *Experimental procedures* section (n ≥ 4). *C*, the permeability in PAR1-R41A-L45P cells was measured after pretreatment with GRK5 inhibitor (GRK5-IN-2) (20 μM) for 1 h followed by incubation with APC (25 nM) for 4 h and TNFα (10 ng/ml) for 16 h (n ≥ 4). *D*, the same as (*C*) except that APC-mediated barrier-protective effects were measured in response to poly(I:C) (10 μg/ml). *E*, the same as (*C*) except that cells were pretreated with Rac1 inhibitor (NSC23766, 50 μM), followed by incubation with APC (25 nM) for 4 h and TNFα (10 ng/ml) for 16 h (n ≥ 4). *F*, the same as (*E*) except that APC-mediated barrier-protective effects were measured in response to poly(I:C) (10 μg/ml) (n ≥ 4). Data are presented as mean ± SEM. *p* Values were determined by one-way ANOVA, followed by a Bonferroni multiple comparison test. ∗∗*p* < 0.01, ∗∗∗*p* < 0.001, and ∗∗∗∗*p* < 0.0001. APC, activated protein C; EPCR, endothelial protein C receptor; GRK5, GPCR kinase 5; PAR1, protease-activated receptor; TNFα, tumor necrosis factor alpha.

**Figure 7:**
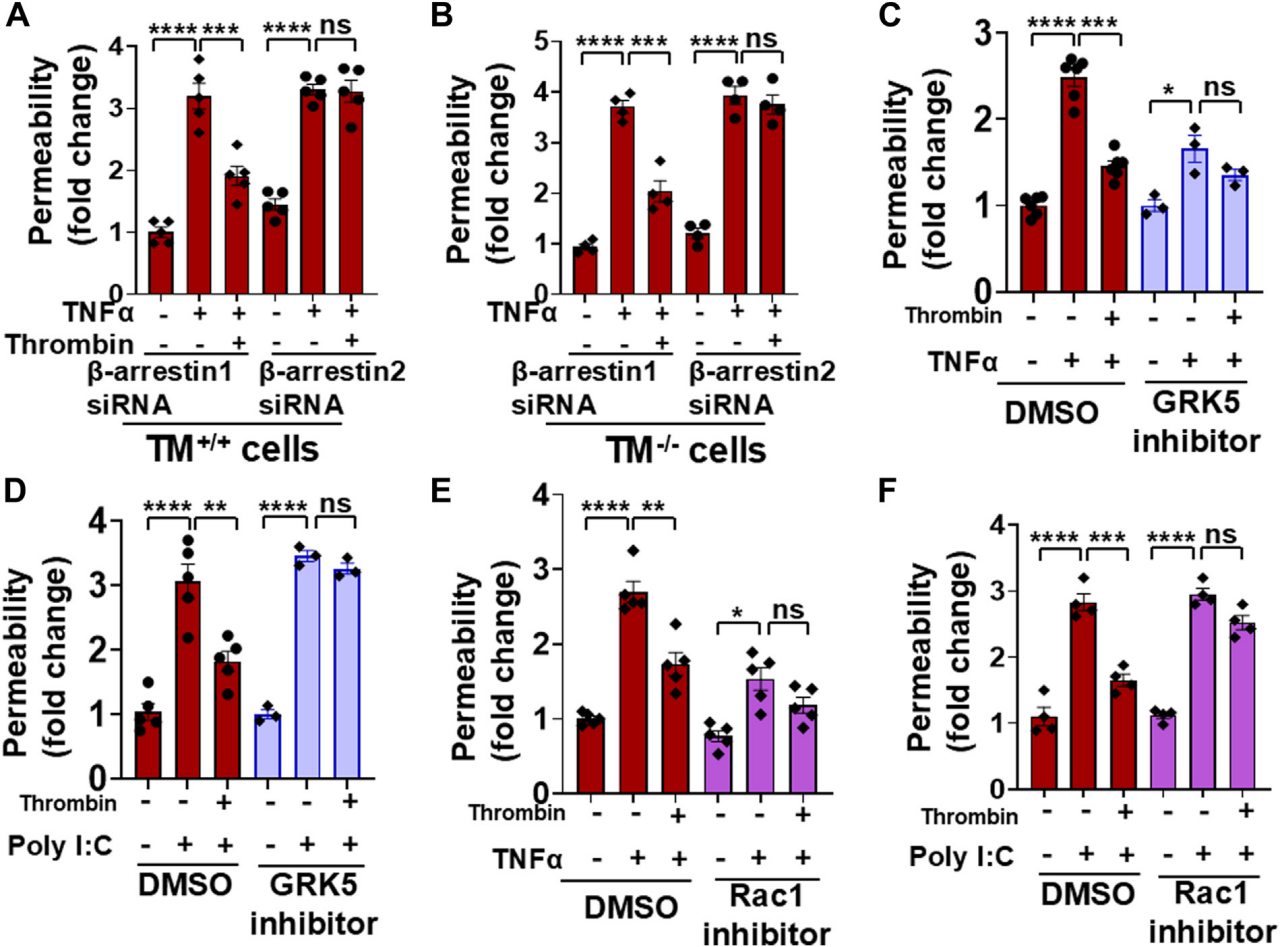
**Analysis of β-arrestin requirement for the cytoprotective thrombin signaling in PAR1-R41A-L45P cells**. TM^+/+^ (*A*) and TM^−/−^ (*B*) cells expressing PAR1-R41A-L45P were transfected with β-arrestin1 or β-arrestin2 siRNA. After 48 h, cells were incubated with thrombin (1 nM) for 4 h followed by TNFα (10 ng/ml) for 16 h, and the permeability was measured as described in the *Experimental procedures* section (n ≥ 4). *C*, the permeability in PAR1-R41A-L45P cells was measured after pretreatment with GRK5 inhibitor (GRK5-IN-2) (20 μM) for 1 h followed by incubation with thrombin (1 nM) for 4 h and TNFα (10 ng/ml) for 16 h (n ≥ 4). *D*, the same as (*C*) except that thrombin-mediated effects were measured in response to poly(I:C) (10 μg/ml). *E*, the same as (*C*) except that cells were pretreated with Rac1 inhibitor (NSC23766, 50 μM), followed by incubation with thrombin (1 nM) for 4 h and TNFα (10 ng/ml) for 16 h (n ≥ 4). *F*, the same as (*E*) except that thrombin-mediated barrier-protective effects were measured in response to poly(I:C) (10 μg/ml) (n ≥ 4). Data are presented as mean ± SEM. *p* Values were determined by one-way ANOVA, followed by a Bonferroni multiple comparison test. ∗*p* < 0.05, ∗∗*p* < 0.01, ∗∗∗*p* < 0.001, and ∗∗∗∗*p* < 0.0001. GRK5, GPCR kinase 5; PAR1, protease-activated receptor 1; TM, thrombomodulin; TNFα, tumor necrosis factor alpha.

We previously demonstrated that both thrombin and APC in the presence of their receptors elicit cytoprotective effects *via* the cleavage of Arg46 through a β-arrestin2-biased PAR1 signaling since the siRNA knockdown of β-arrestin2, but not β-arrestin1, abrogated the protective effects of both proteases in PAR1-R41A cells (2). The results presented in Figure 6 for APC and Figure 7 for thrombin suggest that this is also true for both proteases in Arg46 cleavage–dependent protective signaling in PAR1^−/−^ cells expressing PAR1-R41A-L45P. Thus, the siRNA knockdown of β-arrestin2 but not β-arrestin1 abrogated the Arg46-dependent protective signaling of APC in PAR1-R41A-L45P cells (Fig. 6*A*). The requirement for β-arrestin2-biased signaling was independent of EPCR since its knockdown without blocking EPCR (Fig. 6*A*) or blocking EPCR by a specific anti-EPCR antibody (Fig. 6*B*) yielded the same results. The β-arrestin2-dependent cytoprotective signaling of both APC and thrombin *via* cleavage of Arg46 is reported to be mediated *via* the recruitment of GPCR kinase 5 (GRK5) to the plasma membrane that phosphorylates the cytoplasmic domain of PAR1, thereby preventing the coupling of this domain to a G-protein and instead facilitating its interaction with β-arrestin2, resulting in activation of Rac1, a small GTPase involved in Arg46 cleavage–dependent cytoprotective signaling function of both thrombin and APC (12). In agreement with this mechanism of activation, both specific GRK5 and Rac1 inhibitors abrogated the barrier-protective effect of APC in PAR1-R41A-L45P cells in response to both proinflammatory stimuli TNFα and poly(I:C) (Fig. 6, *C*–*F*). It should be noted that the magnitude of the TNFα-mediated permeability effect was reduced in the presence of both GRK5 and Rac1 inhibitors (Fig. 6, *C* and *E*). This agrees with published data that, both GRK5 and Rac1 inhibitors interfere with the proinflammatory signaling activity of TNFα (13, 14, 15, 16). Thus, in addition to TNFα, we also conducted the permeability assays in the presence of inhibitors in the same endothelial cells activated with poly(I:C) (Fig. 6, *D* and *F*).

The requirement for the β-arrestin2-biased signaling mediated by GRK5 and Rac1 for thrombin activation of PAR1-R41A-L45P was investigated and was found to be the same as APC. Thus, β-arrestin2 was required for signaling by thrombin independent of TM since the siRNA knockdown of β-arrestin2 but not β-arrestin1 abrogated the Arg46-dependent protective signaling by thrombin in both TM^+/+^ and TM^−/−^ cells (Fig. 7, *A* and *B*). Similarly, specific inhibitors of both GRK5 and Rac1 inhibited the barrier-protective effect of thrombin in PAR1-R41A-L45P cells in response to both TNFα and poly(I:C) (Fig. 7, *C*–*F*). Taken together, these results suggest that the receptor-independent and Arg46 cleavage–dependent cytoprotective function of both APC and thrombin is mediated *via* GRK5-dependent PAR1 phosphorylation and β-arrestin2-biased signaling, leading to the activation of Rac1 GTPase in endothelial cells.

### Expression of PAR1 reporter constructs in human embryonic kidney 293 cells and analysis of Arg46 cleavage

**Figure 8:**
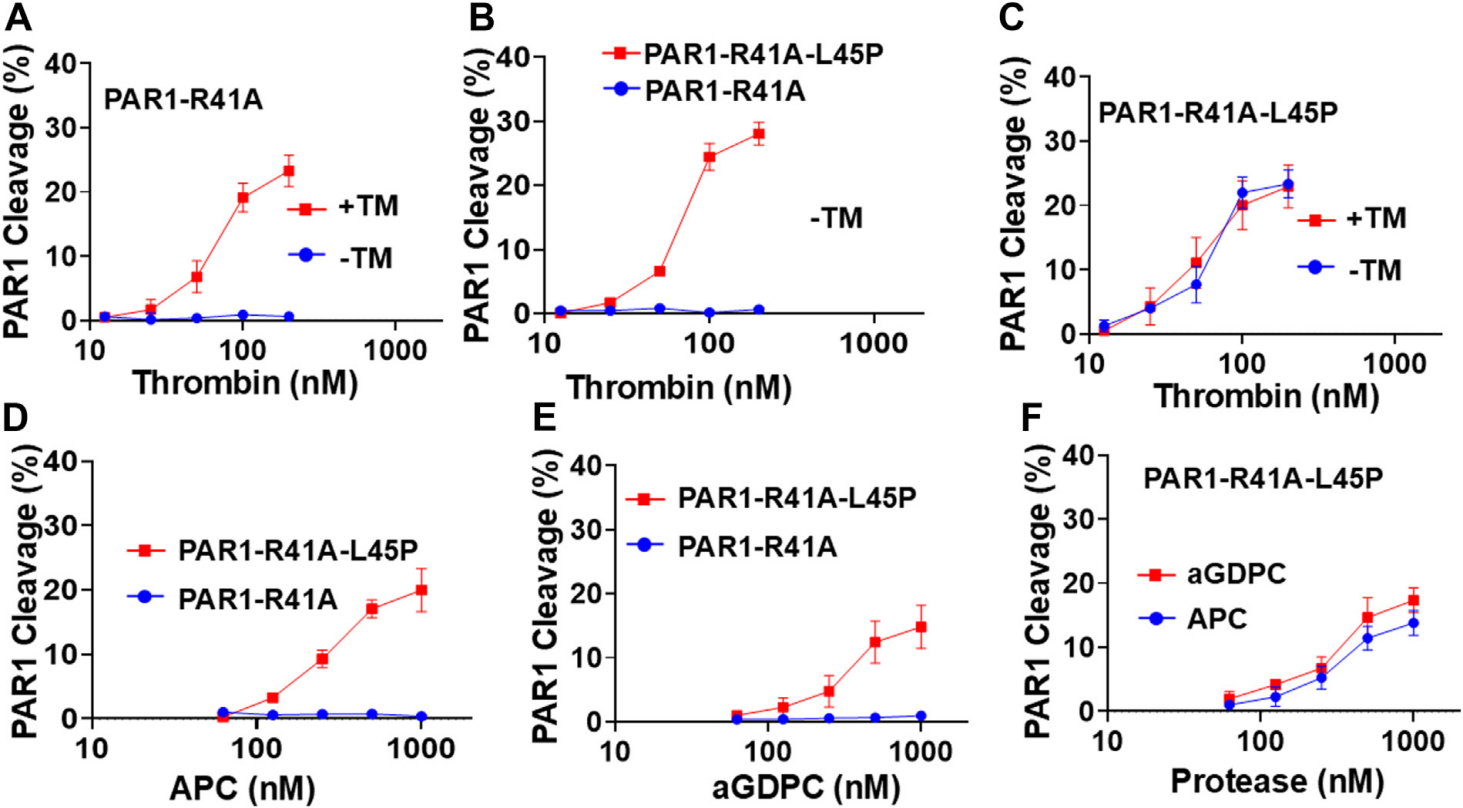
**The efficiency of PAR1-Arg46 cleavage by thrombin and APC**. *A*, stable HEK-293 cells expressing TM and PAR1 reporter construct NLuc-PAR1-R41A-eYFP (*red*) or only expressing the reporter construct (*blue*) were incubated with increasing concentrations of thrombin, and the rate of the cleavage of Arg46 was monitored from the release of luciferase into the cell supernatant as described in the *Experimental procedures* section. The activity of 10 nM thrombin toward Nluc-PAR1-WT-eYFP is presented as 100%. *B*, the same as (*A*) except that the cleavage of both reporter constructs, NLuc-PAR1-R41A-eYFP (*blue*) or NLuc-PAR1-R41A-L45P-eYFP (*red*), was measured in HEK-293 cells not expressing TM. *C*, the same as (*A*) except that the Arg46 cleavage in HEK-293 cells expressing NLuc-PAR1-R41A-L45P-eYFP by thrombin was monitored in the absence (*blue*) or the presence of TM (*red*). *D*, the cleavage of both reporter constructs, NLuc-PAR1-R41A-eYFP (*blue*) or NLuc-PAR1-R41A-L45P-eYFP (*red*), was measured in HEK-293 cells. *E*, the same as (*D*) except that the rate of the cleavage of Arg46 was measured by aGDPC. *F*, the rate of the cleavage of Arg46 in HEK-293 cells expressing Nluc-PAR1-R41A-L45P-eYFP by aGDPC (*red*) and APC (*blue*) was compared. aGDPC, activated Gla-domainless APC; APC, activated protein C; HEK-293, human embryonic kidney 293 cell line; PAR1, protease-activated receptor 1; TM, thrombomodulin.

In a previous study, we used human embryonic kidney 293 (HEK-293) cells expressing a PAR1-Arg46 cleavage reporter construct and demonstrated that neither APC nor thrombin can cleave the Arg46 scissile bond of PAR1 in the absence of their respective cofactors, EPCR and TM, respectively (2). To provide further support for the hypothesis that the P2-Leu45 of PAR1 is inhibitory for the interaction of the P1-Arg46 scissile bond of PAR1 with catalytic pockets of protease in the absence of their cell surface receptors, this residue was replaced with Pro in the Nanoluc-PAR1-R41A-YFP reporter construct (Nanoluc-PAR1-R41A-L45P-YFP) and transferred the constructs to HEK-293 cells, which express neither cell surface receptors, TM or EPCR (2). The reporter constructs were also transferred to HEK-293 cells expressing TM. The thrombin concentration dependence of PAR1 cleavage in HEK-293 cells transfected with the reporter constructs indicated that thrombin can only cleave the Arg46 scissile bond in HEK-293 cells expressing the Nanoluc-PAR1-R41A-YFP reporter construct in the presence of TM (Fig. 8*A*). However, thrombin cleaved the Arg46 site in HEK-293 cells expressing Nanoluc-PAR1-R41A-L45P-YFP in both the presence or the absence of TM (Fig. 8, *B* and *C*). Similarly, in HEK-293 cells expressing either Nanoluc-PAR1-R41A-YFP or Nanoluc-PAR1-R41A-L45P-YFP reporter construct, APC only cleaved the Arg46 scissile bond in the latter L45P mutant construct (Fig. 8*D*). Similarly, aGDPC, which lacks the EPCR-binding Gla domain of APC, cleaved the Arg46 scissile bond of Nanoluc-PAR1-R41A-L45P-YFP but not Nanoluc-PAR1-R41A-YFP cleavage reporter construct (Fig. 8*E*), and both APC and aGDPC exhibited similar cleavage efficiencies (Fig. 8*F*). These results indicate that the P2-Leu45 residue of PAR1 extracellular domain is responsible for the inability of both thrombin and APC to accommodate and cleave the Arg46 scissile bond of PAR1 and that the cofactor functions of EPCR and TM alleviate the inhibitory interactions of this residue with catalytic pockets of these proteases on cell surfaces. The protease concentration dependence of cleavage efficiencies indicates that thrombin cleaves Arg46 of PAR1 in Nanoluc-PAR1-R41A-L45P-YFP reporter construct ∼5- to 10-fold faster than APC independent of a cell surface receptor, and this is consistent with the functional data where thrombin also exhibits a similar extent of higher barrier-protective function endothelial cells expressing the PAR1 constructs (Fig. 1).

## Discussion

In a recent study, we demonstrated that the interaction with TM switches the PAR1 cleavage and signaling specificity of thrombin from a cytopathic to a cytoprotective response by cleaving the Arg46 site of the receptor by a mechanism that was identical to that of APC when the protease binds its endothelial cell surface receptor, EPCR (2). The Arg46-dependent cytoprotective effects of both thrombin and APC required their interaction with their respective cell surface receptors since in their absence both proteases cleaved Arg41 to elicit cytopathic responses in endothelial cells (2). In this study, we investigated the mechanism by which TM and EPCR switch the signaling specificity of proteases from a cytopathic to a cytoprotective response. Since the P1 scissile bond in both cleavage sites is an Arg (P1-R41 and P1-R46), but the P2 residue is a preferred Pro40 and nonpreferred Leu45 for the two sites, we hypothesize that the cell surface receptors may function as cofactors to eliminate the inhibitory interaction of the P2-Leu45 with the catalytic pockets of these proteases. To investigate this hypothesis, we substituted P2-Leu45 of PAR1 with a Pro in an expression vector in which Arg41 of the receptor was replaced with an Ala (PAR1-R41A) so that the proteases can only cleave Arg46 of the receptor (PAR1-R41A-L45P). The protease-dependent signaling specificity of these PAR1 constructs was compared in the absence and presence of the receptors, EPCR and TM. In support of our hypothesis, neither APC nor thrombin exhibited cytoprotective activities in PAR1-R41A cells in the absence of their respective receptors; however, both proteases exhibited normal cytoprotective activities in PAR1-R41A-L45P cells independent of their cell surface receptors.

In all signaling assays described previously, the interaction of thrombin with TM was also required for the barrier-protective and anti-inflammatory function of thrombin in PAR1-R41A cells; however, the protective functions of thrombin in PAR1-R41A-L45P cells were independent of TM. Interestingly, a thrombin mutant (K70D), which is defective for interaction with both the hirudin-like site of PAR1 and TM (11), did not exhibit a barrier-protective effect in normal (TM^+/+^) cells expressing PAR1-R41A but exhibited a WT thrombin-like barrier-protective function in cells expressing PAR1-R41A-L45P. Results with this mutant are very informative since it indicates that the cytoprotective signaling function of thrombin in PAR1-R41A-L45P cells is mediated by a thrombin exosite 1-independent mechanism since the mutation in thrombin-K70D is in exosite 1 of thrombin, and the mutant can bind neither TM nor the hirudin-like site of PAR1.

The other interesting observation of this study was that the receptor-independent cytoprotective signaling of both proteases in PAR1-R41A-L45P cells in response to inflammatory stimuli required the recruitment of β-arrestin2 to the plasma membrane. This was evidenced from the observation that the siRNA knockdown of β-arrestin2 abrogated the protective signaling in PAR1-R41A-L45P cells by both proteases independent of their respective cofactors, EPCR (Fig. 6) or TM (Fig. 7). Since the cytoprotective signaling of thrombin in TM-KO (TM^−/−^) cells expressing PAR1-R41A-L45P through cleavage of Arg46 required β-arrestin2-biased signaling, the results rule out a significant role for the cytoplasmic domains of the receptor in recruiting β-arrestin2 to the plasma membrane. The results suggest that conformational changes, induced in the structure of the membrane spanning and/or the cytoplasmic domain of PAR1 upon protease cleavage of the Arg46 scissile bond, may solely be responsible for the recruitment of β-arrestin2 to initiate a cytoprotective-biased signaling. It is known that biased PAR1 signaling by thrombin and APC is initiated by phosphorylation of the cytoplasmic domain of PAR1 by GRK5, thereby preventing coupling of PAR1 to one of the G-proteins (*i*.*e*., G_12/13_ and G_q_), which leads to the activation of the small GTPase RhoA (2, 12, 17). The phosphorylation of the cytoplasmic domain of PAR1 facilitates the interaction of this domain with β-arrestin2, leading to activation of the Rac1 GTPase and a protective-biased signaling. The observations that the knockdown of β-arrestin2 and inhibition of either GRK5 or Rac1 eliminated the receptor-independent cytoprotective signaling of both APC and thrombin in PAR1-R41A-L45P cells support the hypothesis that the protease cleavage of Arg46 is primarily responsible for the recruitment of signaling molecules required for a β-arrestin2-biased PAR1 signaling in endothelial cells as previously described (1, 2). It is worth noting that in a previous study, we demonstrated that the substitution of the Gla domain of the prothrombin activation intermediate, meizothrombin, with the corresponding domain of protein C renders the mutant protease capable of interacting with EPCR and initiating a cytoprotective effect in endothelial cells (18). Thus, it appears that both EPCR and TM function as protective receptors to maintain the integrity of the vasculature through site-specific activation of PAR1-Arg46 by these proteases. However, during injury and trauma, which can lead to denudation of the endothelium and/or downregulation of these receptors, thrombin initiates proinflammatory responses by binding to hirudin-like sequence of PAR1 and activating the receptor through cleavage of Arg41.

Finally, results derived from the functional data are all supported by the Arg46 cleavage reporter data demonstrating that the cleavage of Arg46 in PAR1 by both APC and thrombin requires the cofactor function of their respective cell surface receptors, EPCR and TM, respectively. The results suggest that the P2-Leu45, as a nonpreferred residue, plays an inhibitory role in allowing the docking of the Arg46 scissile bond into the catalytic pocket of these coagulation proteases and that the cell surface receptors function as cofactors to eliminate the related inhibitory interactions. Since all three receptors, PAR1, TM, and EPCR, are colocalized in the lipid-raft microenvironments of endothelial cells (19), the elimination of the inhibitory interaction by EPCR and TM is made possible by the receptors immobilizing these coagulation proteases on the membrane surface next to PAR1 and aligning their catalytic pockets at a proper distance and orientation to facilitate the docking of the P1-Arg46 scissile bond into the protease catalytic pockets by a mechanism referred to as “substrate presentation” (2). This mechanism of cofactor-dependent catalytic function of coagulation proteases is a recurring theme in the protease-substrate recognition mechanism in the coagulation field (20). Coagulation proteases in general exhibit poor activity toward their target macromolecular substrates primarily because they contain inhibitory nonpreferred residues surrounding the P1-Arg scissile bonds (7, 8). However, interaction of coagulation proteases with their specific cofactors overcomes the inhibitory interactions of nonpreferred residues, thereby facilitating the catalytic reactions at a physiologically relevant rate. The best example is the activation of factors IX and X by factor VIIa, which occurs at a very poor rate and is nearly undetectable in the absence of the cell surface receptor, tissue factor (21). The requirement for cofactors for the normal physiological function of coagulation proteases is an additional step naturally put as a safeguard mechanism for tight regulation of the proteolytic pathways in coagulation and inflammation. The hirudin-like site of PAR1 plays a similar cofactor role in thrombin cleavage of the Arg41 scissile bond; in this case however, the cofactor function is mediated by the negatively charged residue of the hirudin-like site of PAR1 itself, the binding of which to basic residues of the exosite 1 of thrombin dramatically promotes the rate of the cleavage reaction (4, 5, 6). This mechanism of cofactor function is reminiscent of the activation of procofactors V and VIII by thrombin through exosite 1-dependent interaction of the protease with complementary binding sites on these procoagulant cofactors (22).

## Experimental procedures

### Cell culture and reagents

Transformed human umbilical vein endothelial cells (EA.hy926) and HEK-293 cells were obtained from American Type Culture Collection and were maintained in complete Dulbecco's modified Eagle's medium containing 10% fetal bovine serum, 100 μg/ml penicillin, 100 μg/ml streptomycin, 1X HAT supplement (25-046; Mediatech, Inc), and 2 mM l-glutamine. aGDPC and Thrombin K70D mutant were prepared as described (7, 11). HEK-293 cells expressing TM were prepared by lentivirus-based vector transduction system as described (2). The protease-phosphatase inhibitor cocktail, Alexa Flour 488–conjugated goat anti-rabbit IgG (#A11008), NE-PER Nuclear and Cytoplasmic Extraction Reagents (#78833), and Mem-PERTM Plus (#89842) were obtained from Thermo Fisher. PE-conjugated PAR1 antibody (clone ATAP2 [#sc13503] and PE-conjugated mouse isotype control antibody [#sc2866], GAPDH antibody [#sc-47724]) were purchased from Santa Cruz Biotechnology. Anti-EPCR (clone JRK1535) antibody was obtained from Millipore. W146 (S1P_1_ antagonist), NSC 23766 (Rac1 inhibitor) was from Cayman Chemical. GRK-5-IN-2 (GRK-5 inhibitor) was from MedChemExpress. Anti-phospho-VE-cadherin antibody (441144), Lipofectamine 3000 (L3000001), and Lipofectamine RNAiMAX (13778075) were obtained from Invitrogen. Phospho Src antibody (6943T), Src antibody (#2109), VE-cadherin antibody (#2500), anti-Phospho-NF-κB p65 (Ser536) (#3033), anti-NF-κB p65 (#8242), β-arrestin-1 (#12697), β-arrestin-2 (#3857), and β-actin (#4967) antibodies were from Cell Signaling Technology. Human TNFα was from R&D, and poly(I:C) was from InvivoGen.

### Construction of expression vectors

Lentivirus-based PAR1 expression vectors carrying the complementary DNA for WT PAR1 (PAR1-WT), Arg41 to Ala (PAR1-R41A), and Leu45 to Pro (PAR1-R41A-L45P); and lentivirus-based expression vectors carrying the complementary DNA for TM and EPCR were constructed by VectorBuilder, Inc as described (2). The cleavage reporter vectors include PAR1-WT, PAR1-R41A, and PAR1-R41A-L45P; all carrying an N-terminal NanoLuc luciferase, and C-terminal enhanced YFP tags (Nluc-PAR1-WT-eYFP, Nluc-PAR1-R41A-eYFP, and Nluc-PAR1-R41A-L45P-eYFF) were constructed as described (23).

### Re-expression of PAR1 variants in PAR1 knockout endothelial cells

PAR1-knockout (PAR1^−/−^) EA.hy926 endothelial cells were prepared as per the CRISPR–Cas9 methods as described (2). Cells were maintained in complete Dulbecco's modified Eagle's medium containing 10% fetal bovine serum, 100 μg/ml penicillin, 100 μg/ml streptomycin, 1X HAT supplement, and 2 mM l-glutamine. For re-expression of PAR1 constructs (PAR1-WT, PAR1-R41A, and PAR1-R41A-L45P), subconfluent PAR1^−/−^ cells (70–80%) were transduced with lentivirus particles carrying PAR1 derivatives as described (2). After 5 days, cells were selected and maintained in selection medium containing G418 (400 μg/ml) and puromycin (1 μg/ml). TM-knockout (TM^−/−^) EA.hy926 cells were prepared by CRISPR–Cas9 methods as described (10).

### Expression of reporters and measurement of cleavage efficiencies

The cleavage reporter vectors, which all carried a G418 resistant gene, were transfected to HEK-293 cells using Lipofectamine-3000 according to the manufacturer’s protocol. The stable G418-resistant clones were identified and sorted by flow cytometry, based on YFP. FACS-sorted cells were expanded, and several vials for each clone were prepared and frozen in liquid nitrogen for future use. The cleavage reporter constructs were also transfected by HEK-293 cells expressing either TM or EPCR, which were prepared by transducing lentivirus particles carrying either TM or EPCR as described (2). The ability of proteases to cleave the exodomain of PAR1 at different cleavage sites was measured by a luciferase assay employing the Nano-Glo Luciferase assay kit (#N1110) as described (23). Briefly, subconfluent WT HEK-293 cells and HEK-293 cells expressing either TM or EPCR were transfected with the cleavage reporter expression vectors; Nluc-PAR1-WT-eYFP, Nluc-PAR1-R41A-eYFP, and Nluc-PAR1-R41A-L45P-eYFP. The luciferase assay was performed on cells cultured in 48-well assay plates, incubated at 37 ^o^C for 48 h, and after washing with Hank’s balanced salt solution (HBSS) (with calcium and magnesium); the monolayers were treated with different concentrations of thrombin and APC in HBSS. After 15 min of protease incubation, 50 μl of cell supernatants were collected in white 96-well plates for the luciferase assay. The substrate solution of Nano-Glo Luciferase Assay kit was diluted 10-fold with HBSS buffer, and 20 μl of the diluted substrate solution was added to the cell supernatants. Luminescence from the luciferase activity was measured using microplate luminometer according to the manufacturer’s instructions. All experiments were performed in duplicates and repeated multiple times, and data were plotted as mean ± SEM. The concentration of the cell surface–cleaved luciferase was determined from a standard curve provided in the kit.

### Permeability assay

Cell permeability was assessed by spectrophotometric measurement of the flux of Evans blue–bound albumin across functional cell monolayer by a modified two-compartment chamber model as described (2, 10, 12). Briefly, PAR1^−/−^ cells carrying different PAR1 derivatives were plated (2 × 10^5^/well) in transwell plates (3 μm pore size and 12-mm diameter) for 2 days. The confluent monolayers were then incubated with different concentrations of APC (1.2–50 nM) or thrombin (0.025–5 nM) in serum-free media containing 0.5% bovine serum albumin (BSA) for 4 h followed by stimulation of cells by TNFα (10 ng/ml) or poly(I:C) (10 μg/ml) for 16 h. Fresh growth medium was added to the lower chamber, and the media in the upper chamber were replaced with Evans blue–BSA (0.67 mg/ml) (Sigma). After 10 min, the absorbance at 650 nm was measured in the lower chamber. For siRNA transfection, endothelial cells (1 × 10^5^/well) were plated in transwell plates (3-μm pore size and 12-mm diameter) and grown overnight. Then the cells were transfected with β-arrestin-1 siRNA (Dharmacon) (5′-CAUAGAACUUGACACAAAU-3′), β-arrestin-2 siRNA (Dharmacon) (5′-GGACCGCAAAGUGUUUGUG-3′), EPCR siRNA (5′-GUGGACGGCGAUGUUAAUUACTT-3′), and control siRNA (Invitrogen; #12935-200) using Lipofectamine-RNAiMAX (#13778075) according to the manufacturer’s instruction. After 48 h of siRNA treatment, cells were treated with different concentrations of APC or thrombin as described. All experiments were repeated at least three times, and results are expressed as fold change in permeability (ratio of treated samples to untreated controls) and represented as mean ± SEM.

### SDS-PAGE and Western blotting

Cell lysates were prepared from confluent cells using lysis buffer (50 mM Tris [pH 7.4], 150 mM NaCl, 0.5% Triton X-100, 0.5% Nonidet P-40, 5 mM EDTA, and protease-phosphatase inhibitor cocktail). Cytoplasmic and nuclear fractions (NE-PER Nuclear and Cytoplasmic Extraction Reagents; #78833) were prepared according to the manufacturer’s protocol. All samples were extracted in cell lysis buffer and boiled in loading buffer with 5% β-mercaptoethanol and resolved on 7.5% to 10% SDS-polyacrylamide gels. The protein samples were transferred to nitrocellulose membrane and incubated with respective antibodies followed by horseradish peroxidase–linked anti-rabbit IgG or anti-mouse IgG. Protein bands were detected using an ECL substrate as described (2).

### Flow cytometry

The cell surface expression of PAR1 in PAR1-R41A and PAR1-R41A-L45P expressing endothelial cells was analyzed by PE-conjugated PAR1 (ATAP2) antibody, and the expression of EPCR was analyzed by anti-EPCR antibody (JRK1535) followed by Alexa Fluor 488–conjugated anti-mouse antibody as described (2). The cell surface expression of receptors was detected using FACS Celesta flow cytometer, and data were analyzed using FlowJo software (Becton, Dickinson and Company).

### Immunofluorescence

Endothelial cells were treated with APC or thrombin in serum-free media containing 0.5% BSA for 4 h followed by stimulation of cells by TNFα (10 ng/ml) for 1 h. Following treatments, cells were fixed in 4% paraformaldehyde and permeabilized in 0.2% Triton X-100/PBS, followed by blocking for 1 h with normal goat serum. For monitoring p65 nuclear translocation, cells were incubated with specific anti-NF-κB p65 antibody overnight at 4 °C, followed by Alexa Fluor 488–conjugated goat anti-rabbit IgG for 1 h as described (2). Cells were mounted in 4′,6-diamidino-2-phenylindole containing antifade mounting media (H-1500-10), and photomicrographs were obtained using a Nikon C2 Confocal Microscope.

### Statistical analysis

Data are presented as mean ± SEM from ≥3 independent experiments. Normal distribution of the data was tested through Shapiro–Wilk test. Data between multiple groups were analyzed by one-way ANOVA followed by Bonferroni multiple comparison test using GraphPad Prism 10.4.2 (GraphPad Software, Inc).

## Data availability

All data are to be shared upon request. Contact information: Alireza R. Rezaie: Ray-Rezaie@OMRF.ORG.

## Conflict of interest

The authors declare that they have no conflicts of interest with the contents of this article.
